# Integrating Bulk RNA Sequencing and CRISPR-Cas9 Screening to Identify Proliferation-Related Genes for Prognostic Stratification in Breast Cancer

**DOI:** 10.7150/jca.113348

**Published:** 2025-06-12

**Authors:** Guixin Wang, Junming Cao, Yuxin Zhu, Shuo Wang, Yingxi Li, Yue Yu, Yao Tian, Xuchen Cao, Xin Wang

**Affiliations:** 1 The First Department of Breast Cancer, Key Laboratory of Cancer Prevention and Therapy, Tianjin's Clinical Research Center for Cancer, National Clinical Research Center for Cancer, Key Laboratory of Breast Cancer Prevention and Therapy, Tianjin Medical University Cancer Institute and Hospital, Tianjin Medical University, Tianjin, 300060, China.; 2 Tianjin Medical University, Tianjin, 300070, China.; 3 Immunology Department, Key Laboratory of Immune Microenvironment and Disease (Ministry of Education), Tianjin Medical University, Tianjin, 300070, China.; 4 Department of General Surgery, Tianjin Medical University General Hospital, Tianjin Key Laboratory of Precise Vascular Reconstruction and Organ Function Repair, Tianjin General Surgery Institute, Tianjin, 300052, China.

**Keywords:** breast cancer, tumor dependency, CRISPR-Cas9, bulk-RNA sequencing, cell proliferation

## Abstract

Breast cancer has become one of the most common malignant tumors in women. Although the emergence of molecular typing has greatly improved the prognosis of breast cancer patients, some patients still face drug resistance, recurrence and metastasis. At present, the development of effective biomarkers is still an important direction of breast cancer research. This study aims to provide new ideas for individualized treatment of breast cancer by identifying new biomarkers and constructing models to predict the prognosis of breast cancer patients. In this study, seven tumor-dependent genes associated with tumor proliferation were identified through the combined analysis of bulk-RNA sequencing and CRISPR-CAS9, and the mechanism of their potential promotion of tumor proliferation was initially analyzed. Immune infiltration analysis suggested these genes may be associated with the formation of immunosuppressive microenvironment. In addition, we constructed a gene signature based on seven genes that can predict prognostic risk in patients with breast cancer. The group with higher signature scores was associated with more GATA3 somatic mutations. Finally, we screened potential drugs suitable for high-risk groups to improve their outcomes. Our study provides potential therapeutic targets as well as individualized treatment strategies for breast cancer.

## Introduction

Breast cancer (BRCA) has emerged as one of the most prevalent and lethal malignancies among women globally in recent years 1-3. According to 2020 global cancer statistics, over 2.26 million new cases and approximately 685,000 deaths were reported, with incidence rates continuing to rise annually 4. Although advancements in medical technologies, including surgical interventions, radiotherapy, chemotherapy, targeted therapies, and endocrine treatments, have significantly improved patient prognosis 5, the high intra- and inter-tumoral heterogeneity of breast cancer leads to variable therapeutic sensitivities 6. Therefore, identifying novel prognostic biomarkers and potential therapeutic targets remains imperative to expand intervention strategies for breast cancer patients.

The goal of the Dependency Map (DepMap) database, integrating CRISPR screening data from hundreds of cancer cell lines, systematically identifies genes essential for tumor cell survival and proliferation across cancer types, serving as a pivotal tool for discovering cancer dependency genes 7. Genes identified through CRISPR screenings exhibit high clinical translational value. For instance, Xiao et al. 8 analyzed DepMap CRISPR-Cas9 data to investigate olaparib sensitivity-related gene expression variations, revealing that *NLRP4* upregulation enhances olaparib resistance in pancreatic cancer. Similarly, Li et al. 9 identified three proliferation-associated genes in triple-positive breast cancer using CRISPR-Cas9 and constructed a prognostic prediction model. These studies highlight the potential of DepMap-based dependency analyses in guiding precision oncology. Cross-validation with databases like The Cancer Genome Atlas (TCGA) may further strengthen the clinical relevance of candidate genes.

In this study, we identified seven tumor dependency-associated genes by integrating CRISPR-Cas9 whole-genome knockout data with TCGA transcriptomic profiles. These genes were significantly correlated with poor prognosis, low CD8 T cell infiltration, and high M2 macrophage infiltration in breast cancer. Furthermore, a tumor dependency signature constructed from these genes effectively stratified patient outcomes, with the high-dependency subgroup demonstrating enrichment in *GATA3* mutations. Finally, potential therapeutic agents targeting high-dependency patients were screened to guide personalized treatment. Our findings provide novel insights and candidate targets for breast cancer therapy.

## Materials and Methods

### Data acquisition and preprocessing

**Figure 1:**
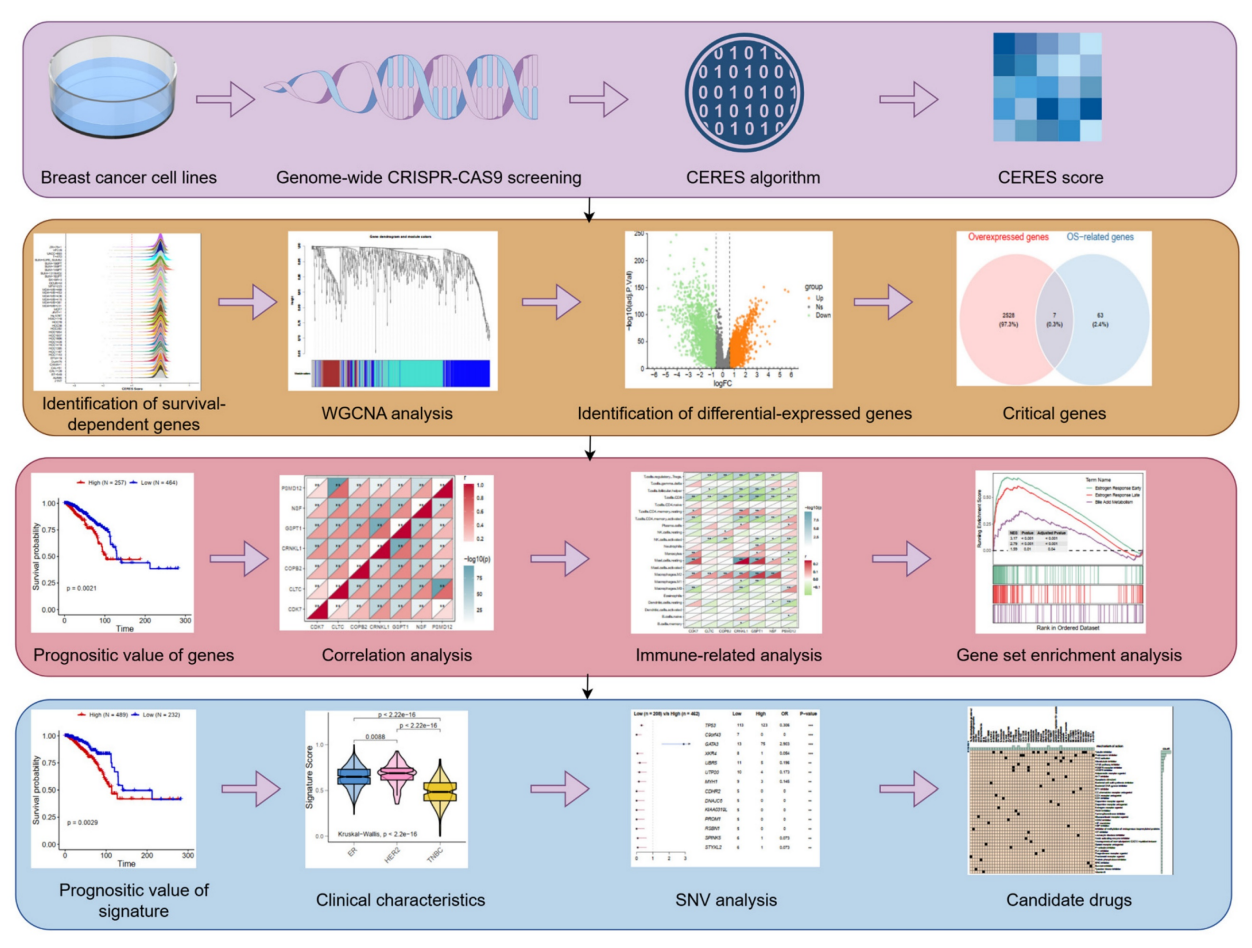
** Schematic design of the study.** WGCNA, Weighted Gene Co-Expression Network Analysis; SNV, single nucleotide variants.

The workflow of this study is illustrated in Figure 1. The DepMap database provides genome-wide CRISPR-Cas9 knockout data across multiple cancer cell lines. CERES matrices from 42 breast cancer cell lines post-CRISPR-Cas9 knockout were downloaded and processed. To identify tumor proliferation dependency genes, the following criteria were applied: 1) Genes meeting CERES scores < -1 in ≥75% of breast cancer cell lines; 2) Genes with average CERES scores < -1 across all breast cancer cell lines. Lower CERES scores indicate stronger tumor proliferation dependency. Filtered genes were preliminarily designated as proliferation-associated genes for downstream analyses.

Transcriptomic profiles and somatic mutation data of breast cancer were retrieved from the TCGA database via the 'TCGAbiolinks' (v2.26.0) package 10, while clinical data were obtained from the XENA database. Transcriptomic data were processed using TPM (transcripts per million)-normalized expression matrices to minimize biases from sequencing depth and gene length. Inclusion criteria for samples were: 1) Availability of estrogen receptor* (ER)*, progesterone receptor* (PR)*, and Erb-B2 receptor tyrosine kinase 2 (*HER2*) status confirmed by immunohistochemistry (IHC) or immunofluorescence; 2) Complete survival information (overall survival time and status); 3) Transcriptomic data availability. After quality control, 721 TCGA-BRCA samples were retained for subsequent analyses.

### WGCNA analysis

A total of 698 proliferation-associated genes from 721 samples were included in weighted gene co-expression network analysis (WGCNA) through 'WGCNA' (v1.72-1) package 11. Outlier samples were excluded prior to constructing an unsigned topological matrix. The soft thresholding power was set to 5, with a minimum module size of 30 genes. Three gene modules were ultimately identified.

### Survival and differential expression analysis

Univariate Cox regression analysis was performed to evaluate the prognostic value of each module. Differential expression analysis between 113 normal breast tissues and 721 tumor samples was conducted using the 'limma' (3.54.2) package 12. Genes with log2(fold change) > 0.585 and P < 0.05 were defined as differentially expressed. Intersection analysis between proliferation-associated genes and differentially expressed genes was performed by 'venn' (v1.11) package. Kaplan-Meier survival curves were generated to assess associations between gene expression levels and overall survival (P < 0.05 considered statistically significant). Of note, 'survminer' (v0.4.9) package was used to determine the best cut-off value of grouping.

### Immune infiltration and correlation analysis

CIBERSORTx 13 was employed to estimate immune cell infiltration in TCGA-BRCA samples using the LM22 signature matrix as a reference. Spearman correlation analysis was performed to evaluate relationships between the seven proliferation-associated genes and immune cell abundance, immune checkpoint molecule expression, and inter-gene correlations (P < 0.05 considered statistically significant).

### ssGSEA and GSEA

A gene signature score comprising the seven candidate genes was established using single-sample gene set enrichment analysis (ssGSEA) via 'GSVA' (v1.46.0) package. Subsequently, the signature scores were standardized. Samples were stratified into high- and low-proliferation groups based on median signature scores. 'genesets.v2023.1.hallmark.Hs' was obtained from GSEA database. Differentially expressed genes identified via 'limma' were input into gene set enrichment analysis (GSEA) with the " genesets.v2023.1.hallmark.Hs" gene sets as reference. Pathways with P < 0.05 were considered statistically enriched.

### Clinical feature and somatic mutation analysis

Kaplan-Meier analysis evaluated the prognostic value of the gene signature. Associations between signature scores and molecular subtypes were assessed via Kruskal-Wallis test, while correlations with clinical features (T stage, M stage, tumor stage) were analyzed using Wilcoxon test (P < 0.05). The 'maftools' (v2.14.0) package was utilized to compare genomic heterogeneity between high- and low-signature groups. Fisher's exact test identified differential somatic mutations, and mutation landscapes of the top 15 mutated genes were visualized (P < 0.05).

### Enrichment analysis and drug identification

The top 1500 genes in the high-signature group underwent functional enrichment analysis using the 'clusterProfiler' (v4.6.2) package 14. The CAMP database 15 screened candidate therapeutic compounds by comparing top 100 genes from high- and low-signature groups (version 1.0). Over 2400 compounds were scored, with lower scores indicating stronger inhibitory effects. The 50 compounds with the lowest scores were selected as potential therapeutics for the high-signature group.

### Statistics

All of the statistical analyses are described above and analyzed by R (v4.2.2).

## Results

### Identification of tumor proliferation-associated genes

**Figure 2:**
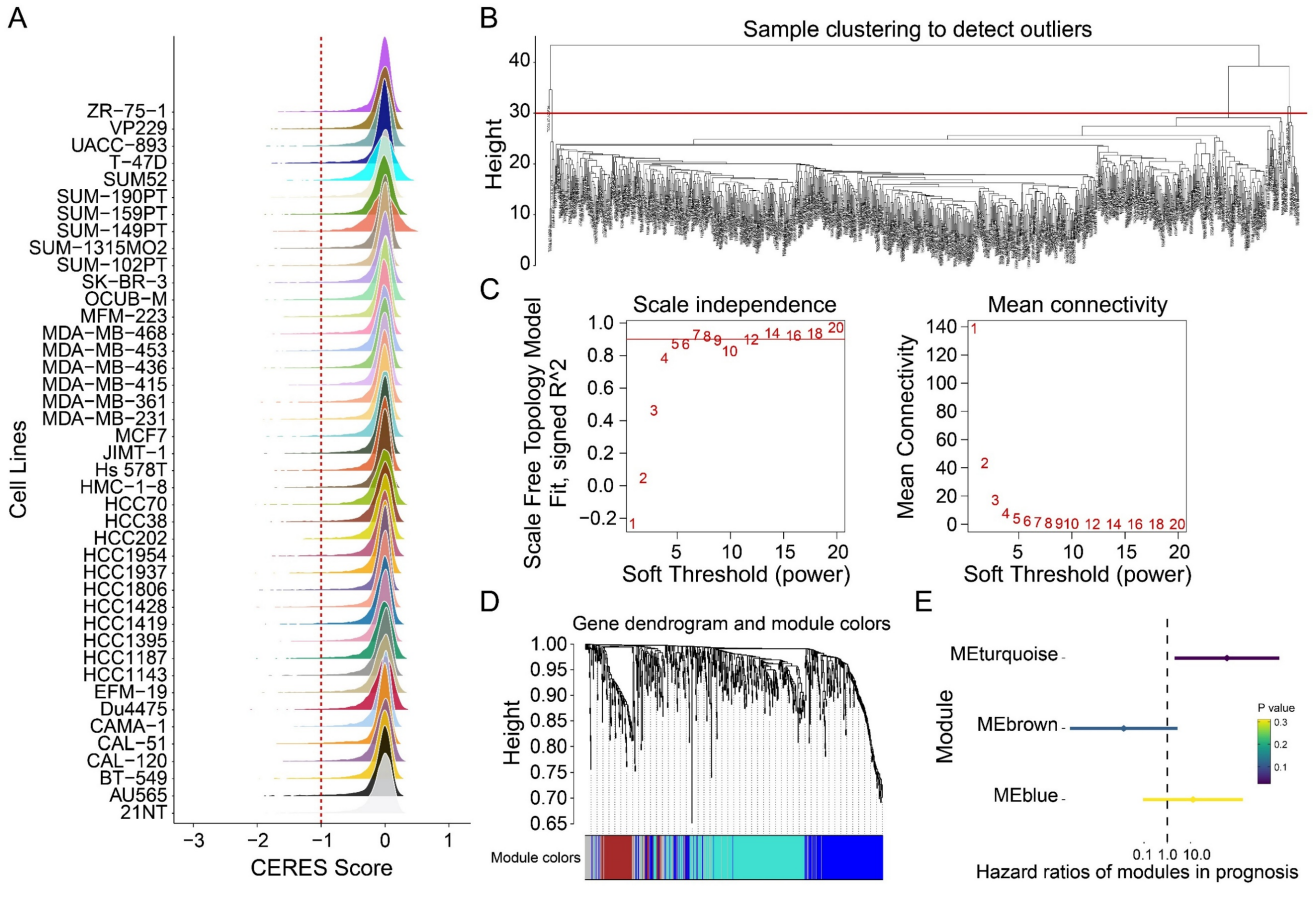
** Identification of tumor dependency-related genes. (A)** The peak map showing the CERES score distribution of 42 breast tumor cell lines. **(B)** Sample clustering. **(C)** Scale-free fit index for various soft-thresholding powers. Mean connectivity for various soft-thresholding powers **(D)** Dendrogram of all differentially expressed genes clustered based on dissimilarity measure (1-TOM). **(E)** The forest map showing the prognosis of the module.

Genome-wide CRISPR-Cas9 knockout data from 42 breast cancer cell lines were analyzed using the CERES algorithm, yielding a CERES matrix of 17,386 genes. Through stringent filtering (CERES score < -1 in ≥75% of cell lines), 698 tumor proliferation-associated genes were identified (Figure 2A). Weighted gene co-expression network analysis (WGCNA) was subsequently performed on 721 samples to identify genes linked to overall survival. After hierarchical clustering and removal of 17 outlier samples (Figure 2B), an unsigned topological overlap matrix was constructed with a soft thresholding power of 5 (scale-free R² = 0.9; Figure 2C). Three gene modules were defined (minimum module size: 30 genes; Figure 2D, Table S1). Univariate Cox regression revealed that the blue module (N=275) significantly correlated with poor prognosis (P<0.05; Figure 2E). Taken together, 275 genes were prioritized as tumor proliferation-associated candidates for further investigation.

### Prognostic validation of seven tumor proliferation-associated genes

**Figure 3:**
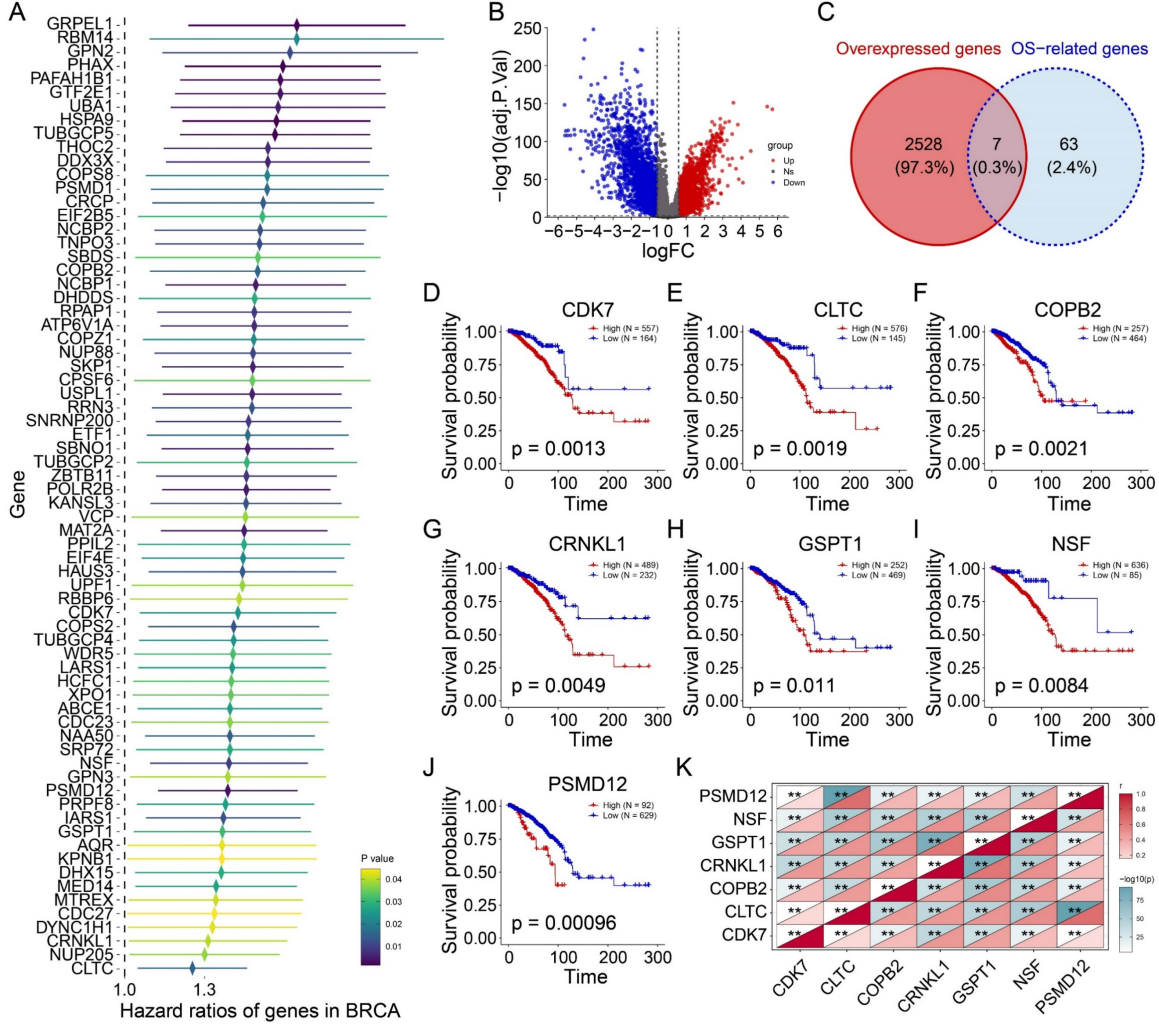
** The prognostic value of candidate genes. (A)** The forest map showing the prognosis of 42 tumor proliferation-related genes.** (B)** Volcanic plot exhibiting differential expressed genes (DEGs).** (C)** Venn plot showing the intersection of two gene sets.** (D-J)** Kaplan-Meiers curves of CDK7 **(D)**, CLTC **(E)**, COPB2 **(F)**, CRNKL1 **(G)**, GSPT1 **(H)**, NSF **(I)**, and PSMD12 **(J)**.** (K)** Heatmap showing the correlations among candidate genes. **P<0.01.

Univariate Cox regression of the 275 candidate genes identified 42 genes significantly associated with adverse outcomes (Figure 3A). Differential expression analysis between 721 tumor and 113 normal tissues revealed 2,535 upregulated genes (log2FC > 0.585, P<0.05; Figure 3B). Intersection analysis identified seven genes (*CDK7*, *CLTC*, *COPB2*, *CRNKL1*, *GSPT1*, *NSF*, *PSMD12*) that were both upregulated and prognostically significant (Figure 3C). Kaplan-Meier analysis confirmed their strong association with reduced overall survival (Figures 3D-J; *CDK7*: P=0.0013; *CLTC*: P=0.0019; *COPB2*: P=0.0021; *CRNKL1*: P=0.0049; *GSPT1*: P=0.011; *NSF*: P=0.0084; *PSMD12*: P=0.00096). Notably, these genes exhibited a strong co-expressed pattern (Figure 3K). Taken together, these findings suggest that high expression of these seven genes in tumors is associated with poor prognosis and may be potential prognostic markers and therapeutic targets for BRCA.

### Correlation between candidate tumor proliferation-related genes and immune infiltration

**Figure 4:**
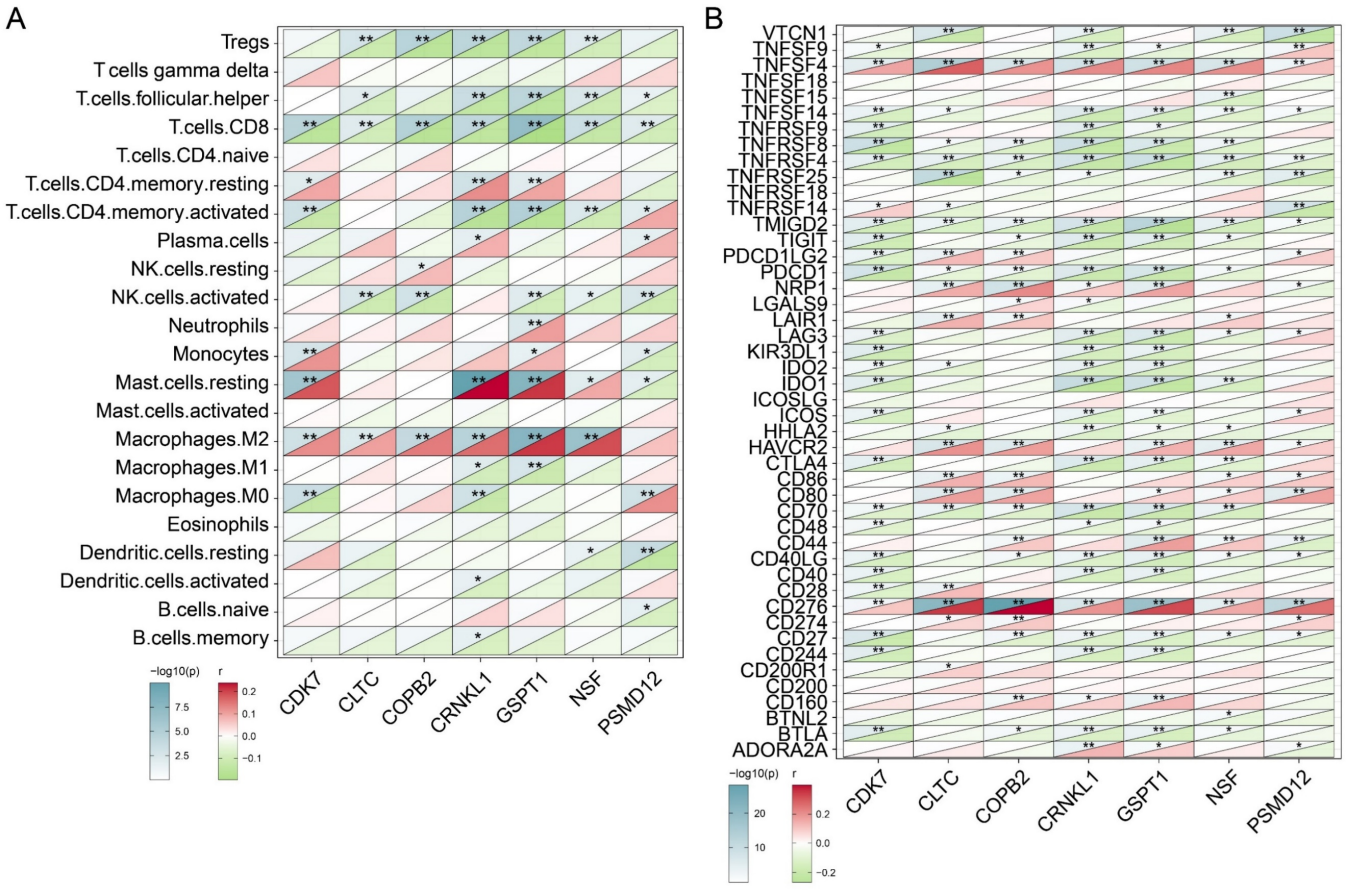
** Exploring the correlations between tumor dependency genes and immune infiltration. (A)** Heatmap exhibiting the correlations between candidate genes and 22 distinct immune cell infiltration. **(B)** Heatmap exhibiting the correlations between candidate genes and immune checkpoints. *P< 0.05, **P<0.01.

The above findings have initially confirmed the prognostic role of the seven genes we identified, and we next analyzed their association with immune infiltration. CIBERSORTX was used to assess the levels of infiltration of various immune cells in BRCA samples and to analyze its correlation with the expression levels of seven genes. As shown in Figure 4A, we observed that the expression level of these seven genes was significantly negatively correlated with the infiltration level of CD8 T cells (P<0.01). Besides *CDK6* (P<0.01), *CLTC* (P<0.01), *COPB2* (P<0.01), *CRNKL1* (P<0.01), *GSPT1* (P<0.01), *NSF* (P<0.01) showed significant positive correlation with the abundance of M2 macrophages. The results suggested that the infiltration level of these genes may be related to the formation of immunosuppressive microenvironment of tumors. Subsequently, we analyzed the correlation of these seven genes with the level of immune checkpoint molecular expression. As shown in Figure 4B, the expression levels of *TNFSF4* and *CD276* were significantly positively correlated with expression levels of the above candidate genes. In contrast, the expression levels of *TNFRSF4* and *TMIGD2* were negatively correlated with the expression levels of these genes. These findings indicated that the immune checkpoint molecules *TNFSF4* and *CD276* may benefit patients with high levels of tumor proliferation genes. In a word, these analyses initially revealed subtle links between the genes we identified that are associated with tumor proliferation and immune cells and immune checkpoint molecules.

### Identification of potential signaling pathways of candidate tumor proliferation genes

**Figure 5:**
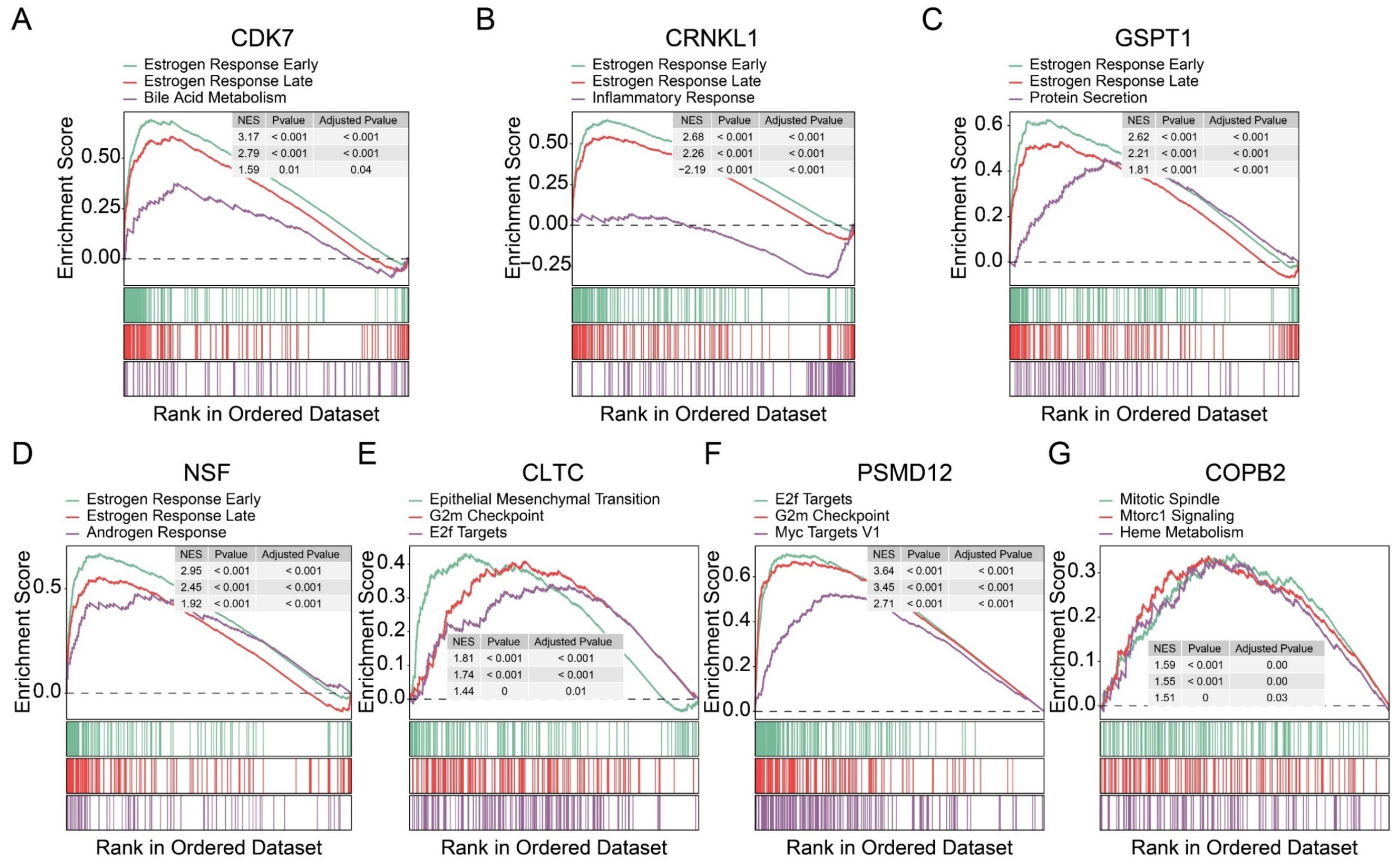
** Exploration of potential mechanism of 7 tumor dependency genes. (A-G)** Gene set enrichment analyses of CDK7 **(A)**, CRNKL1 **(B)**, GSPT1 **(C)**, NSF **(D)**, CLTC **(E)**, PSMD12 **(F)**, and COPB2 **(G)**.

To further identify the pathways associated with these proliferation-related genes, we performed a gene set pathway analysis for each gene separately. Interestingly, we found that the expression levels of *CDK7* (Figure 5A), *CRNKL1* (Figure 5B), *GSPT1* (Figure 5C), and *NSF* (Figure 5D) were significantly correlated with the estrogen pathway. The estrogen pathway has been proved to be a classic pathway to maintain the proliferation of breast cancer cells.* CLTC* (Figure 5E) and *PSMD12* (Figure 5F) were significantly correlated with G2M checkpoint and E2F target channels. The expression level of *COPB2* was also significantly correlated with the mitotic spindle (Figure 5G) pathway. These findings support that these genes are involved in tumor proliferation. At the same time, these results also preliminarily reveal the possible mechanism of action of these genes.

### Construction of a signature related to tumor proliferation

**Figure 6:**
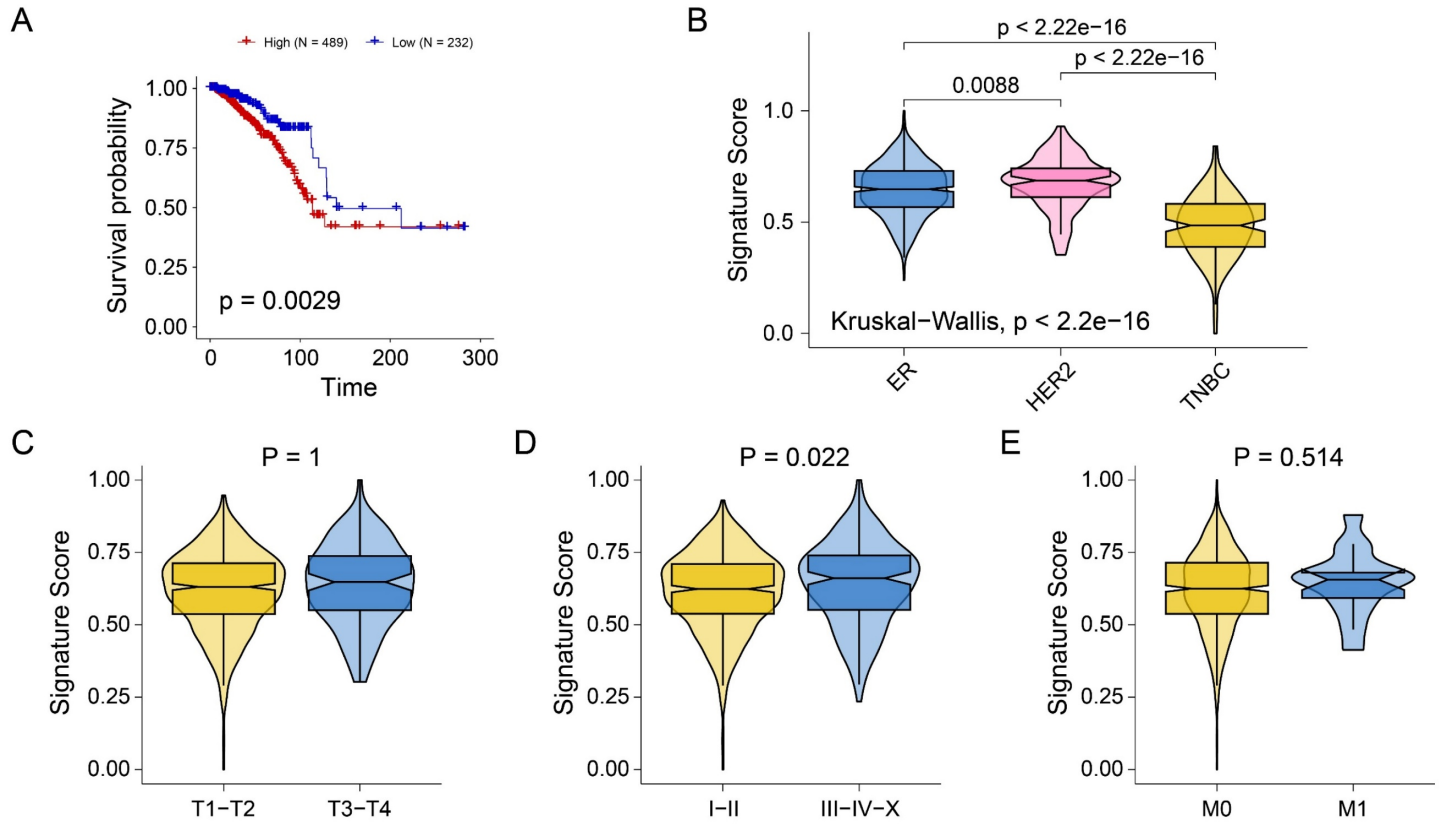
** Construction of tumor proliferation-related gene signature. (A)** Kaplan-Meiers curves showing the prognostic value of signature. **(B-E)** Boxplots showing the signature score in molecular subtypes **(B)**, T stage **(C)**, tumor stage **(D)**, and M stage **(E)**.

The above analysis preliminarily revealed the role of seven genes in prognosis and immune infiltration. Next, we attempted to construct a gene tag based on seven gene compositions to assess individualized tumor proliferation status. The ssGSEA algorithm was used to infer the proliferation-related scores of each BRCA sample according to the expression levels of seven genes. As shown in Figure 6A, signature scores were significantly associated with poor prognosis in BRCA patients (P=0.0029). Interestingly, we observed that signature scores were significantly higher in ER and HER2 breast cancers than in TNBC (Figure 6B). The results showed consistency with the results of previous pathway analysis. In addition, no significant difference in signature scores was observed across stage T (Figure 6C) and stage M (Figure 6E). However, the signature scores in stages I and II were significantly lower than those in stages III-IV-X (Figure 6D). The results indicated that the signature score may be primarily driven by pathways more active in hormone receptor-positive or HER2 subtypes. These findings implied that signature scores are associated with breast cancer prognosis, molecular typing, and tumor stage, and may serve as a novel clinical stratification tool.

### Correlation between signature score and somatic mutations

**Figure 7:**
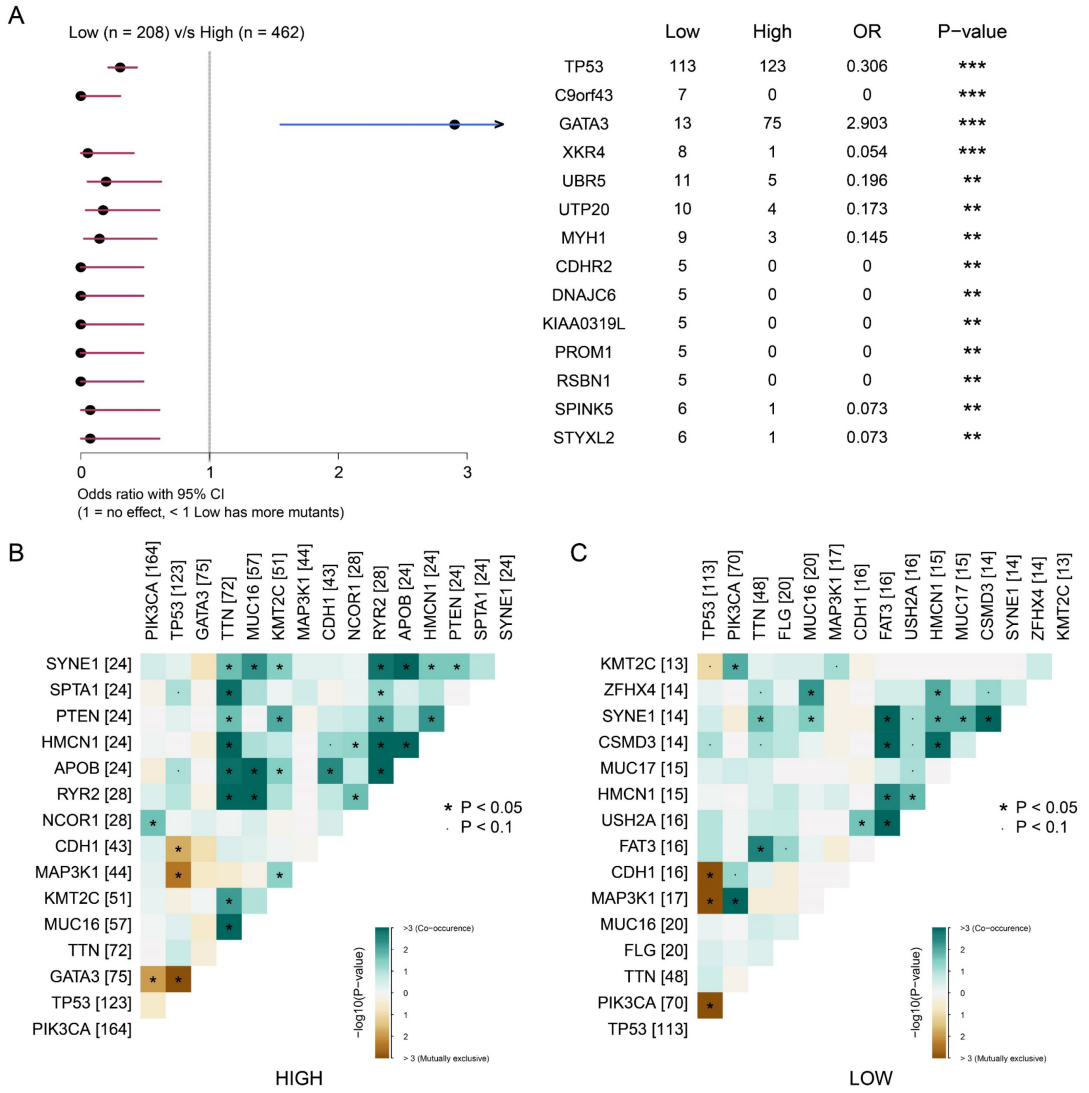
** The focal somatic mutations were associated with the signature score based on tumor dependency genes. (A)** Forest plot demonstrating focal somatic single nucleotide variant (SNV) and copy-number alteration (CNA) frequencies that are significantly different between patients in the high- and low- signature groups. OR>1, higher SNV frequency for patients in the high signature group. OR<1, higher SNV frequency in low signature group. **P<0.01, ***P<0.001 **(B-C)** Landscape of gene co-mutations in patients with high- **(B)** and low- **(C)** signature scores.

We then explored the correlation between somatic mutations and tumor proliferation. We divided the samples into high signature group and low signature group according to the signature score and the best cut-off value. As shown in Figure 7A, we observed that the high signature group was significantly associated with somatic mutations in *GATA3*. The low signature group was significantly associated with somatic mutations of *TP53, C9orf43, XKR4* and other genes. This suggests that somatic mutations of *GATA3* may be related to BRCA proliferation. Next, we analyze the relationship between signature scores and mutation patterns. As shown in Figure 7B-C, the group with a high signature score showed a more obvious co-mutation pattern than the group with a low signature score, while no significant difference was found in co-rejection between the two groups. These results reveal a potential link between tumor proliferation-associated signature and somatic cells, and suggest that mutations in *GATA3*, as well as co-mutation patterns, may be associated with breast cancer proliferation.

### Drug identification based on proliferation-related signature

**Figure 8:**
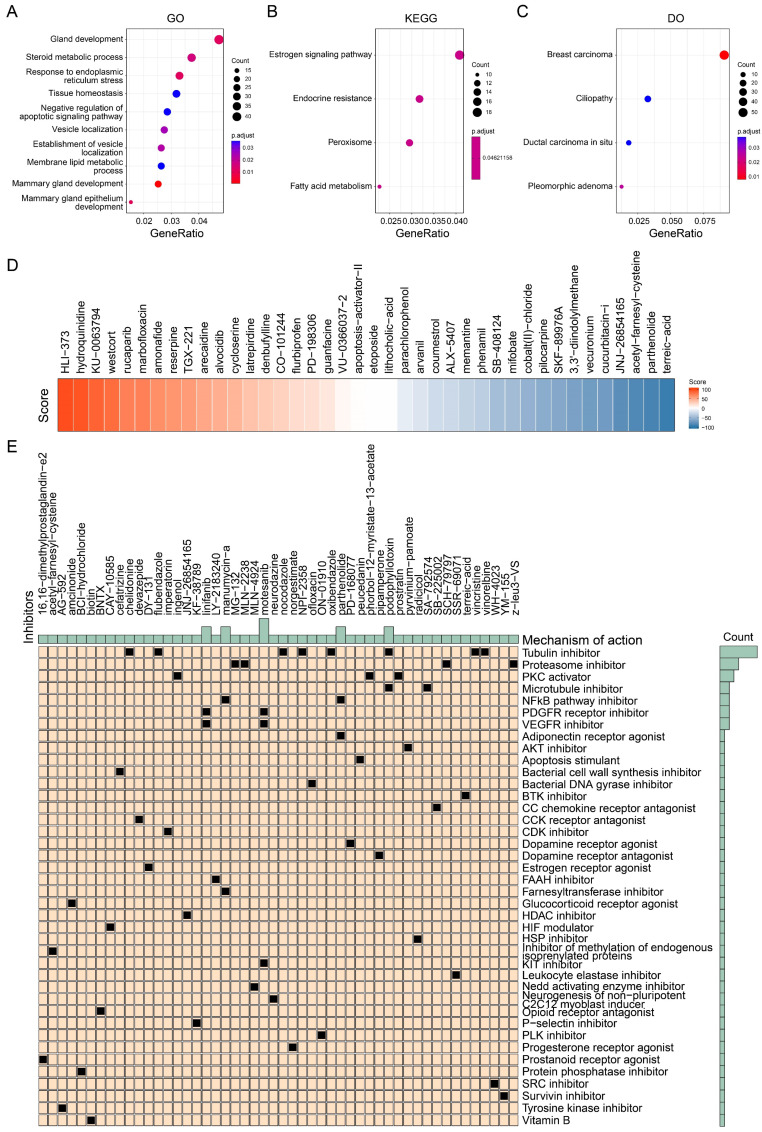
** Identification of potential drugs suitable for high signature score patients. (A)** Gene ontology enrichment analysis of top 1500 genes of high signature score patients. **(B)** KEGG enrichment analysis of top 1500 genes of high signature score patients. **(C)** Disease ontology analysis of top 1500 genes of high signature score patients. **(D)** Heatmap showing the several drugs and their score for examples. **(E)** The potential drugs identified for high signature score patients.

We further analyzed the pathways associated with the signature scores to screen potential drugs. We analyzed the difference between the group with a high signature score and the group with a low signature score according to the previous grouping results, and extracted the top 1500 genes in the group with a high signature score for enrichment analysis. As shown in Figure 8A, GO analysis showed that these genes were associated with the negative regulation of apoptosis signaling pathways and breast development. KEGG analysis showed that these genes were associated with the estrogen signaling pathway and endocrine resistance (Figure 8B). DO analysis showed that these genes were most associated with breast cancer (Figure 8C). These findings are consistent with the results we obtained above. Because of the poor prognosis in the group with a high signature score, we further screened drugs for patients in the group with a high signature score. More than 2400 compounds and their scores were obtained from the CMAP database. As shown in Figure 8D, some of the drugs and their scores were showed. It should be noted that the lower the score, the closer the compound's action is to that of an inhibitor. Subsequently, we screened the 50 compounds with the lowest scores as drug candidates for patients with high signature scores (Figure 8E, Table S2). In summary, we screened drugs that are potentially suitable for patients with high tumor proliferation signature scores, providing a new idea for individualized treatment of BRCA.

## Discussion

In this study, by integrating CRISPR-CAS9 functional genomics (DEPMAP database) and TCGA multi-omics data, seven key genes (*CDK7, CLTC, COPB2, CRNKL1, GSPT1, NSF* and *PSMD12*) that are closely related to the proliferation and prognosis of breast cancer were systematically identified. A gene signature based on these genes were constructed. The signature can not only effectively distinguish the survival risk of patients, but also significantly associated with the tumor immune microenvironment, molecular typing and somatic mutation characteristics, providing a novel intervention strategy for the individualized treatment of breast cancer.

In this study, seven genes were found to be significantly overexpressed in breast cancer and associated with poor prognosis in patients, suggesting that they may act as oncogenic genes to drive tumor progression. For example, *CDK7* is a core kinase in cell cycle regulation, which can promote tumor proliferation and maintain tumor stemness by activating *CDK-RB-E2F*, *YAP*, and other mechanisms, and has been confirmed as a therapeutic target for many cancers 16, 17. As a proteasome subunit, *PSMD12* is involved in the regulation of protein degradation, and its elevated expression inhibits apoptosis signaling pathways to maintain the survival of breast cancer cells 18. In addition, *COPB2* has been reported to promote proliferation in triple-negative breast cancer 19. Long et al. 20 demonstrated that the absence of *GSPT1* inhibits the proliferation and migration of colon cancer cells in vitro. These reports are consistent with our findings. It is worth noting that these seven genes are significantly co-expressed in breast cancer, and whether these genes form a complex network to promote breast cancer progression remains to be verified by further experiments. Immune infiltration is an important indicator of tumor response to treatment 21, 22. Thus, we analyzed the correlations between gene expression and immune infiltration. The expression levels of the seven genes were negatively correlated with CD8+ T cell infiltration, but positively correlated with M2 macrophage infiltration, suggesting that they may promote immune escape by reshaping the immunosuppressive microenvironment. M2 macrophages inhibit T cell activity by secreting *IL-10* and TGF-β, while promoting angiogenesis and metastasis 23. In addition, the gene signature scores were significantly positively correlated with immune checkpoint molecules *CD276* (*B7-H3*) and *TNFSF4* (*OX40L*), further supporting the existence of an immunosuppressive microenvironment. CD276 has been reported to be significantly negatively associated with immune infiltration of CD8+ T cells in breast cancer 24. The *OX40/OX40L* (*TNFSF4*) signaling pathway has also been reported to promote T cell activation and immune stimulation 25. These findings provide a theoretical basis for the combination of targeting tumor proliferation genes and immune checkpoint inhibitors.

Further analysis showed that the constructed seven-gene signature was significantly overexpressed in ER+/HER2+ subtypes and correlated with advanced tumor stage, suggesting that it may be used as a potential prognostic marker for hormone receptor-positive breast cancer. The group with high signature scores was enriched in estrogen signaling pathways and G2M checkpoints, which is consistent with the classical mechanism of estrogen-dependent proliferation in ER+ breast cancer 26. Notably, co-mutation patterns were more common in the high-signature group than in the low-signature group, suggesting a potential correlation between tumor proliferation characteristics and somatic co-mutation. These findings provide a new perspective for the comprehensive assessment of the proliferation drivers of breast cancer cells. Finally, we screened drugs suitable for patients with high signature scores, providing new ideas for individualized treatment and combination therapy for breast cancer patients.

Some limitations of the study also need to be clarified. First, CRISPR-CAS9 screening data are based on only 42 breast cancer cell lines, which may not fully reflect the heterogeneity of clinical tumors. Second, the prognostic value of the gene signature needs to be validated in prospective cohorts. Third, the sample size of bulk RNA cohort is limited, and more external datasets need to be included to reduce the bias of analyses. Additionally, the oncogenic mechanisms and drug sensitivity of candidate genes still need to be confirmed by in vivo and in vitro experiments. Future studies could be combined with single-cell sequencing technology to analyze the spatial heterogeneity of the gene signature in the tumor microenvironment, and could use organoid models to verify the individualized efficacy of drugs.

## Supplementary Material

Supplementary tables.
