# Supplementary material for: Integrating Bulk RNA Sequencing and CRISPR-Cas9 Screening to Identify Proliferation-Related Genes for Prognostic Stratification in Breast Cancer
Source: J Cancer. 2025 Jun 12;16(9):2800–11. doi: 10.7150/jca.113348 (PMC12244016; doi:10.7150/jca.113348)
Supplement: Supplementary file 1 — Supplementary tables. [file jcav16p2800s1.pdf]

| Table S1. The genes in distinct modules | |
|---|---|
| gene | color |
| AARS1 | blue |
| ABCE1 | turquoise |
| ABCF1 | turquoise |
| ACTL6A | blue |
| AHCTF1 | turquoise |
| AKIRIN2 | blue |
| ALG11 | turquoise |
| ALG1L | grey |
| ANAPC1 | turquoise |
| ANAPC10 | grey |
| ANAPC11 | grey |
| ANAPC2 | grey |
| ANAPC4 | turquoise |
| ANAPC5 | turquoise |
| ANKLE2 | turquoise |
| AQR | turquoise |
| ARCN1 | turquoise |
| ARIH1 | turquoise |
| ARL 2.00 | brown |
| ATP1A1 | turquoise |
| ATP2A2 | turquoise |
| ATP5MF | grey |
| ATP6V0B | grey |
| ATP6V0C | grey |
| ATP6V1A | turquoise |
| ATP6V1B2 | turquoise |
| ATP6V1E1 | grey |
| ATP6V1F | grey |
| ATP6V1G1 | turquoise |

| | |
|---|---|
| AURKA | blue |
| AURKB | blue |
| BANF1 | blue |
| BANP | turquoise |
| BIRC5 | blue |
| BRF1 | grey |
| BRF2 | turquoise |
| BUB1B | blue |
| BUB3 | turquoise |
| BUD23 | blue |
| BUD31 | blue |
| BYSL | blue |
| C1orf131 | turquoise |
| CACTIN | grey |
| CARS1 | blue |
| CCNA2 | blue |
| CCNK | turquoise |
| CCT2 | blue |
| CCT3 | blue |
| CCT4 | blue |
| CCT5 | blue |
| CCT6A | blue |
| CCT7 | blue |
| CCT8 | blue |
| CDC123 | blue |
| CDC16 | turquoise |
| CDC20 | blue |
| CDC23 | turquoise |
| CDC27 | turquoise |
| CDC37 | brown |
| CDC45 | blue |

| | |
|---|---|
| CDC5L | turquoise |
| CDC6 | blue |
| CDC7 | blue |
| CDC73 | turquoise |
| CDCA8 | blue |
| CDK1 | blue |
| CDK7 | turquoise |
| CDK9 | grey |
| CDT1 | blue |
| CENATAC | grey |
| CENPK | blue |
| CENPN | blue |
| CENPW | blue |
| CFAP298 | grey |
| CHAF1A | blue |
| CHAF1B | blue |
| CHD4 | brown |
| CHEK1 | blue |
| CHERP | turquoise |
| CHMP2A | grey |
| CHMP6 | grey |
| CIAO3 | grey |
| CKAP5 | blue |
| CLNS1A | grey |
| CLP 1 | blue |
| CLTC | turquoise |
| CNIH4 | blue |
| CNOT3 | grey |
| COPA | turquoise |
| COPB1 | turquoise |
| COPB2 | turquoise |

| | |
|---|---|
| COPG1 | grey |
| COPS2 | turquoise |
| COPS5 | grey |
| COPS6 | grey |
| COPS8 | turquoise |
| COPZ1 | turquoise |
| CPSF1 | grey |
| CPSF2 | turquoise |
| CPSF3 | blue |
| CPSF4 | blue |
| CPSF6 | turquoise |
| CRCP | turquoise |
| CRNKL1 | turquoise |
| CSE1L | blue |
| CTCF | blue |
| CTDP1 | grey |
| CWC22 | blue |
| DAD1 | grey |
| DARS1 | blue |
| DBR1 | turquoise |
| DCTN5 | turquoise |
| DDB1 | turquoise |
| DDX10 | blue |
| DDX18 | turquoise |
| DDX20 | turquoise |
| DDX3X | turquoise |
| DDX41 | turquoise |
| DDX42 | turquoise |
| DDX46 | turquoise |
| DDX47 | turquoise |
| DDX49 | blue |

| | |
|---|---|
| DDX54 | turquoise |
| DDX56 | blue |
| DHDDS | turquoise |
| DHX15 | turquoise |
| DHX16 | turquoise |
| DHX37 | turquoise |
| DHX8 | turquoise |
| DNAJC17 | grey |
| DNAJC8 | turquoise |
| DNM2 | turquoise |
| DONSON | blue |
| DTL | blue |
| DTYMK | blue |
| DUT | blue |
| DYNC1H1 | turquoise |
| DYNC1I2 | turquoise |
| DYNLRB1 | grey |
| ECD | turquoise |
| EEF1A1 | brown |
| EEF1G | grey |
| EEF2 | brown |
| EEF2KMT | turquoise |
| EFTUD2 | turquoise |
| EIF1AD | blue |
| EIF1AX | turquoise |
| EIF2B1 | turquoise |
| EIF2B2 | turquoise |
| EIF2B3 | blue |
| EIF2B4 | grey |
| EIF2B5 | turquoise |
| EIF2S1 | turquoise |

| | |
|---|---|
| EIF2S2 | blue |
| EIF2S3 | turquoise |
| EIF3A | turquoise |
| EIF3B | blue |
| EIF3D | brown |
| EIF3F | brown |
| EIF3G | brown |
| EIF3I | grey |
| EIF4A3 | blue |
| EIF4E | turquoise |
| EIF5 | turquoise |
| EIF6 | blue |
| ELOB | grey |
| ELOC | grey |
| EPRS1 | turquoise |
| ERCC3 | turquoise |
| ERH | grey |
| ESPL1 | blue |
| ESS2 | blue |
| ETF1 | turquoise |
| EXOSC4 | grey |
| EXOSC6 | blue |
| FARSA | blue |
| FARSB | blue |
| FAU | brown |
| FBL | brown |
| FBXO5 | blue |
| FCF1 | turquoise |
| FIP1L1 | turquoise |
| FTSJ3 | turquoise |
| GARS1 | blue |

| | |
|---|---|
| GBF1 | turquoise |
| GEMIN5 | turquoise |
| GGTLC2 | grey |
| GINS1 | blue |
| GINS2 | blue |
| GINS4 | blue |
| GNL2 | blue |
| GNL3 | turquoise |
| GPN1 | blue |
| GPN2 | turquoise |
| GPN3 | turquoise |
| GPS1 | blue |
| GRPEL1 | turquoise |
| GSPT1 | turquoise |
| GTF2A2 | grey |
| GTF2B | turquoise |
| GTF2E1 | turquoise |
| GTF2E2 | grey |
| GTF2F2 | grey |
| GTF2H1 | turquoise |
| GTPBP4 | blue |
| H2AC12 | blue |
| H2AC16 | grey |
| H2AC17 | grey |
| H2BC11 | blue |
| H2BC15 | grey |
| H2BC4 | grey |
| H2BC5 | grey |
| H2BC6 | grey |
| H3C13 | grey |
| HARS1 | turquoise |

| | |
|---|---|
| HAUS1 | blue |
| HAUS3 | turquoise |
| HAUS5 | blue |
| HAUS6 | turquoise |
| HAUS7 | grey |
| HAUS8 | blue |
| HCFC1 | turquoise |
| HEATR1 | turquoise |
| HINFP | turquoise |
| HMGCS1 | turquoise |
| HNRNPC | turquoise |
| HNRNPK | turquoise |
| HNRNPL | turquoise |
| HSPA5 | grey |
| HSPA9 | turquoise |
| HSPD1 | blue |
| HSPE1 | blue |
| IARS1 | turquoise |
| IFITM3 | grey |
| IGBP1 | grey |
| IK | turquoise |
| IMP3 | brown |
| INCENP | blue |
| INTS1 | grey |
| INTS11 | grey |
| INTS3 | turquoise |
| INTS4 | turquoise |
| INTS8 | blue |
| INTS9 | turquoise |
| ISCU | grey |
| ISY1 | blue |

| | |
|---|---|
| KANSL3 | turquoise |
| KARS1 | blue |
| KAT8 | turquoise |
| KIF11 | blue |
| KIF23 | blue |
| KIN | blue |
| KPNB1 | turquoise |
| KRR1 | turquoise |
| KRT8 | grey |
| LARS1 | turquoise |
| LCE5A | grey |
| LONP1 | blue |
| LRR1 | blue |
| LSM 2.00 | blue |
| LSM 3.00 | blue |
| LSM 4.00 | blue |
| LSM 5.00 | blue |
| LSM 6.00 | blue |
| LSM 7.00 | brown |
| LSM 8.00 | turquoise |
| LTO1 | grey |
| LUC7L3 | turquoise |
| MAD2L1 | blue |
| MAK16 | turquoise |
| MARS1 | blue |
| MASTL | blue |
| MAT2A | turquoise |
| MCM2 | blue |
| MCM3 | blue |
| MCM4 | blue |
| MCM5 | blue |

| | |
|---|---|
| MCM6 | blue |
| MCM7 | blue |
| MDN1 | turquoise |
| MED11 | grey |
| MED14 | turquoise |
| MED20 | turquoise |
| MED22 | turquoise |
| MED28 | turquoise |
| MED30 | blue |
| MED4 | turquoise |
| MED6 | turquoise |
| MED8 | blue |
| MEPCE | grey |
| MFAP1 | turquoise |
| MMS22L | blue |
| MPHOSPH10 | blue |
| MTBP | blue |
| MTREX | turquoise |
| MYC | brown |
| MZT1 | turquoise |
| NAA50 | turquoise |
| NACA | brown |
| NAPA | turquoise |
| NARS1 | turquoise |
| NCAPD2 | blue |
| NCAPG | blue |
| NCBP1 | turquoise |
| NCBP2 | turquoise |
| NDC80 | blue |
| NEDD1 | turquoise |
| NEDD8 | grey |

| | |
|---|---|
| NFS1 | turquoise |
| NIFK | blue |
| NIP7 | blue |
| NLE1 | turquoise |
| NOL10 | blue |
| NOL6 | turquoise |
| NOP16 | blue |
| NOP56 | blue |
| NOP58 | blue |
| NPLOC4 | blue |
| NRF1 | turquoise |
| NSA2 | brown |
| NSF | turquoise |
| NUDT21 | blue |
| NUF2 | blue |
| NUP133 | turquoise |
| NUP160 | turquoise |
| NUP205 | turquoise |
| NUP214 | turquoise |
| NUP85 | blue |
| NUP88 | turquoise |
| NUP93 | blue |
| NUS1 | blue |
| NUTF2 | blue |
| NVL | turquoise |
| NXF1 | turquoise |
| ORC1 | blue |
| ORC6 | blue |
| PABPN1 | turquoise |
| PAFAH1B1 | turquoise |
| PAM16 | turquoise |

| | |
|---|---|
| PCBP1 | grey |
| PCNA | blue |
| PDCD11 | turquoise |
| PDRG1 | grey |
| PFDN2 | blue |
| PFDN6 | blue |
| PHAX | turquoise |
| PHB | grey |
| PHB2 | brown |
| PHF5A | blue |
| PLK1 | blue |
| PMF1 | brown |
| PMPCA | turquoise |
| PMPCB | turquoise |
| POLA2 | blue |
| POLD1 | blue |
| POLD2 | blue |
| POLD3 | turquoise |
| POLE | turquoise |
| POLE2 | blue |
| POLR1A | turquoise |
| POLR1B | turquoise |
| POLR1C | blue |
| POLR1F | turquoise |
| POLR2B | turquoise |
| POLR2C | blue |
| POLR2D | blue |
| POLR2E | brown |
| POLR2F | blue |
| POLR2G | blue |
| POLR2H | blue |

| | |
|---|---|
| POLR2I | brown |
| POLR2L | brown |
| POLR3A | turquoise |
| POLR3B | turquoise |
| POLR3C | turquoise |
| POLR3F | turquoise |
| POLR3H | turquoise |
| POLR3K | grey |
| POP5 | grey |
| PPIL2 | turquoise |
| PPP1R11 | turquoise |
| PPP1R8 | turquoise |
| PPWD1 | turquoise |
| PRC1 | blue |
| PRELID1 | blue |
| PRELID3B | blue |
| PRIM1 | blue |
| PRMT5 | turquoise |
| PRPF19 | turquoise |
| PRPF31 | grey |
| PRPF38A | blue |
| PRPF38B | turquoise |
| PRPF4 | turquoise |
| PRPF6 | grey |
| PRPF8 | turquoise |
| PSMA1 | blue |
| PSMA2 | turquoise |
| PSMA3 | blue |
| PSMA4 | blue |
| PSMA5 | blue |
| PSMA6 | grey |

| | |
|---|---|
| PSMA7 | blue |
| PSMB1 | blue |
| PSMB2 | blue |
| PSMB3 | grey |
| PSMB4 | blue |
| PSMB5 | blue |
| PSMB6 | grey |
| PSMB7 | grey |
| PSMC1 | turquoise |
| PSMC2 | turquoise |
| PSMC3 | blue |
| PSMC4 | blue |
| PSMC5 | grey |
| PSMC6 | turquoise |
| PSMD1 | turquoise |
| PSMD11 | blue |
| PSMD12 | turquoise |
| PSMD13 | blue |
| PSMD14 | blue |
| PSMD2 | blue |
| PSMD3 | grey |
| PSMD4 | blue |
| PSMD6 | turquoise |
| PSMD7 | blue |
| PSMD8 | blue |
| PSMG3 | blue |
| PUF60 | grey |
| PWP2 | grey |
| QARS1 | brown |
| RABGGTA | grey |
| RABGGTB | turquoise |

| | |
|---|---|
| RACGAP1 | blue |
| RACK1 | brown |
| RAD21 | blue |
| RAE1 | blue |
| RAN | blue |
| RANGAP1 | blue |
| RBBP4 | turquoise |
| RBBP6 | turquoise |
| RBM14 | turquoise |
| RBM17 | blue |
| RBM22 | turquoise |
| RBM25 | turquoise |
| RBM39 | turquoise |
| RBM8A | turquoise |
| RBMX | turquoise |
| RBMX2 | blue |
| RBX1 | grey |
| RCC1 | blue |
| RFC2 | blue |
| RFC3 | blue |
| RFC5 | blue |
| RIOK2 | turquoise |
| RNF113A | grey |
| RNGTT | blue |
| RNPC3 | turquoise |
| RNPS1 | grey |
| RPA1 | turquoise |
| RPA2 | turquoise |
| RPA3 | turquoise |
| RPAIN | turquoise |
| RPAP1 | turquoise |

| | |
|---|---|
| RPAP2 | turquoise |
| RPL10A | brown |
| RPL11 | brown |
| RPL12 | brown |
| RPL13 | brown |
| RPL13A | brown |
| RPL14 | brown |
| RPL15 | brown |
| RPL18 | brown |
| RPL18A | brown |
| RPL19 | brown |
| RPL21 | brown |
| RPL23 | brown |
| RPL23A | brown |
| RPL24 | brown |
| RPL26 | brown |
| RPL27 | brown |
| RPL27A | brown |
| RPL3 | brown |
| RPL30 | brown |
| RPL31 | brown |
| RPL32 | brown |
| RPL35 | brown |
| RPL35A | brown |
| RPL36 | brown |
| RPL37 | brown |
| RPL37A | brown |
| RPL38 | brown |
| RPL4 | brown |
| RPL5 | brown |
| RPL6 | brown |

| | |
|---|---|
| RPL7 | brown |
| RPL8 | brown |
| RPLP0 | brown |
| RPLP1 | brown |
| RPLP2 | brown |
| RPS10 | grey |
| RPS11 | brown |
| RPS12 | brown |
| RPS13 | brown |
| RPS15 | brown |
| RPS15A | brown |
| RPS16 | brown |
| RPS18 | brown |
| RPS19 | brown |
| RPS2 | brown |
| RPS20 | brown |
| RPS21 | brown |
| RPS23 | brown |
| RPS24 | brown |
| RPS25 | brown |
| RPS27A | brown |
| RPS29 | brown |
| RPS3 | brown |
| RPS4X | brown |
| RPS5 | brown |
| RPS6 | brown |
| RPS7 | brown |
| RPS8 | brown |
| RPS9 | brown |
| RPSA | brown |
| RRM1 | blue |

| | |
|---|---|
| RRM2 | blue |
| RRN3 | turquoise |
| RSL1D1 | turquoise |
| RUVBL1 | blue |
| RUVBL2 | brown |
| SACM1L | turquoise |
| SAP18 | grey |
| SAP30BP | blue |
| SARS1 | grey |
| SART1 | brown |
| SART3 | turquoise |
| SBDS | turquoise |
| SBNO1 | turquoise |
| SCFD1 | turquoise |
| SDE2 | turquoise |
| SEC13 | blue |
| SEC61A1 | turquoise |
| SEC61G | blue |
| SF1 | turquoise |
| SF3A1 | turquoise |
| SF3A2 | brown |
| SF3A3 | blue |
| SF3B1 | turquoise |
| SF3B2 | blue |
| SF3B3 | blue |
| SF3B4 | blue |
| SF3B5 | grey |
| SF3B6 | blue |
| SFPQ | turquoise |
| SGO1 | blue |
| SKP1 | turquoise |

| | |
|---|---|
| SLC39A7 | turquoise |
| SLU7 | turquoise |
| SMC1A | turquoise |
| SMC2 | turquoise |
| SMC4 | turquoise |
| SMG1 | turquoise |
| SMG5 | turquoise |
| SMR3B | grey |
| SMU1 | turquoise |
| SNAPC1 | turquoise |
| SNAPC2 | brown |
| SNAPC4 | turquoise |
| SNAPC5 | turquoise |
| SNRNP200 | turquoise |
| SNRNP25 | grey |
| SNRNP27 | turquoise |
| SNRNP35 | grey |
| SNRNP70 | grey |
| SNRPA1 | blue |
| SNRPB | blue |
| SNRPC | blue |
| SNRPD1 | blue |
| SNRPD2 | brown |
| SNRPF | blue |
| SNU13 | grey |
| SNW1 | turquoise |
| SOD1 | grey |
| SON | turquoise |
| SPC24 | blue |
| SPC25 | blue |
| SPCS2 | grey |

| | |
|---|---|
| SPDL1 | blue |
| SPOUT1 | grey |
| SRBD1 | turquoise |
| SRCAP | turquoise |
| SRP54 | turquoise |
| SRP72 | turquoise |
| SRP9 | turquoise |
| SRSF1 | turquoise |
| SRSF2 | turquoise |
| SRSF3 | turquoise |
| SRSF7 | turquoise |
| SS18L2 | grey |
| SSRP1 | blue |
| SSU72 | brown |
| STX5 | grey |
| SUPT16H | turquoise |
| SUPT5H | turquoise |
| SUPT6H | turquoise |
| SYMPK | turquoise |
| TAF6 | turquoise |
| TANGO6 | grey |
| TARS1 | blue |
| TCP1 | blue |
| THOC1 | turquoise |
| THOC2 | turquoise |
| THOC3 | blue |
| THOC5 | blue |
| THOC7 | grey |
| TICRR | blue |
| TIGD1 | blue |
| TIMELESS | blue |

| | |
|---|---|
| TINF2 | turquoise |
| TIPIN | blue |
| TNPO3 | turquoise |
| TOMM40 | blue |
| TONSL | blue |
| TOP 1.00 | turquoise |
| TOP2A | blue |
| TOPBP1 | turquoise |
| TRAPPC3 | blue |
| TRAPPC5 | grey |
| TRAPPC8 | turquoise |
| TRMT112 | brown |
| TRRAP | turquoise |
| TSG101 | turquoise |
| TSR2 | grey |
| TTC27 | turquoise |
| TUBA1B | blue |
| TUBB | blue |
| TUBG1 | grey |
| TUBGCP2 | turquoise |
| TUBGCP3 | turquoise |
| TUBGCP4 | turquoise |
| TUBGCP5 | turquoise |
| TUT1 | grey |
| TXNL4A | blue |
| U2AF2 | grey |
| U2SURP | turquoise |
| UBA1 | turquoise |
| UBA2 | blue |
| UBA52 | brown |
| UBE2I | turquoise |

| | |
|---|---|
| UBL5 | grey |
| UBR4 | turquoise |
| UBTF | turquoise |
| UFD1 | blue |
| UPF1 | turquoise |
| UPF2 | blue |
| URI1 | turquoise |
| USP36 | blue |
| USP39 | blue |
| USP5 | grey |
| USPL1 | turquoise |
| UTP11 | blue |
| UTP15 | turquoise |
| UTP20 | turquoise |
| UTP4 | blue |
| VARS1 | turquoise |
| VCP | turquoise |
| VIRMA | grey |
| VPS25 | turquoise |
| VPS28 | grey |
| WARS1 | blue |
| WBP11 | turquoise |
| WDR12 | blue |
| WDR33 | turquoise |
| WDR43 | blue |
| WDR5 | turquoise |
| WDR70 | turquoise |
| WDR74 | brown |
| WDR75 | blue |
| WDR77 | blue |
| WDR82 | turquoise |

| | |
|---|---|
| WEE1 | turquoise |
| XAB2 | brown |
| XPO1 | turquoise |
| XRCC6 | turquoise |
| YARS1 | blue |
| YJU2 | brown |
| YKT6 | blue |
| YRDC | blue |
| ZBTB11 | turquoise |
| ZMAT2 | turquoise |
| ZMAT5 | grey |
| ZNF131 | turquoise |
| ZNF207 | turquoise |
| ZNF335 | turquoise |
| ZNF830 | turquoise |
| ZNHIT2 | brown |
| ZPR1 | blue |

Table S2 The candidate drugs identified by CMAP

| Name | Score | MOA |
|---|---|---|
| SB-225002 | -77.42 | CC chemokine receptor antagonist |
| ofloxacin | -78.51 | Bacterial DNA gyrase inhibitor |
| norgestimate | -78.99 | Progesterone receptor agonist |
| z-leu3-VS | -79.33 | Proteasome inhibitor |
| imperatorin | -79.73 | CDK inhibitor |
| podophyllotoxin | -79.86 | Microtubule inhibitor, Tubulin inhibitor |
| phorbol-12-myristate-13-acetate | -79.94 | PKC activator |
| cefatrizine | -79.99 | Bacterial cell wall synthesis inhibitor |
| YM-155 | -80.1 | Survivin inhibitor |

| | | |
|---|---|---|
| amcinonide | -80.35 | Glucocorticoid receptor agonist |
| SSR-69071 | -80.47 | Leukocyte elastase inhibitor |
| linifanib | -80.76 | PDGFR receptor inhibitor, VEGFR inhibitor |
| JNJ-26854165 | -81.96 | HDAC inhibitor |
| oxibendazole | -82.54 | Tubulin inhibitor |
| WH-4023 | -83.53 | SRC inhibitor |
| ingenol | -83.82 | PKC activator |
| AG-592 | -83.83 | Tyrosine kinase inhibitor |
| MG-132 | -84.11 | Proteasome inhibitor |
| radicicol | -84.26 | HSP inhibitor |
| chelidonine | -84.46 | Tubulin inhibitor |
| ON-01910 | -84.58 | PLK inhibitor |
| acetyl-farnesyl-cysteine | -86.31 | Inhibitor of methylation of endogenous isoprenylated proteins |
| nocodazole | -87.05 | Tubulin inhibitor |
| 16,16-dimethylprostaglandin-e2 | -87.52 | Prostanoid receptor agonist |
| manumycin-a | -88.1 | Farnesyltransferase inhibitor, NFkB pathway inhibitor |
| SCH-79797 | -88.95 | Proteasome inhibitor |
| pyrvinium-pamoate | -89.49 | AKT inhibitor |
| pipamperone | -90.03 | Dopamine receptor antagonist |
| parthenolide | -90.23 | NFkB pathway inhibitor, Adiponectin receptor agonist |
| vincristine | -90.35 | Tubulin inhibitor |
| MLN-2238 | -90.84 | Proteasome inhibitor |
| MLN-4924 | -90.94 | Nedd activating enzyme inhibitor |
| KF-38789 | -91.26 | P-selectin inhibitor |
| PD-168077 | -91.33 | Dopamine receptor agonist |
| biotin | -91.75 | Vitamin B |
| devazepide | -92.14 | CCK receptor antagonist |
| BNTX | -92.95 | Opioid receptor antagonist |
| LY-2183240 | -93.44 | FAAH inhibitor |
| BCI-hydrochloride | -94.16 | Protein phosphatase inhibitor |
| prostratin | -94.5 | PKC activator |

| | | |
|---|---|---|
| DY-131 | -94.8 | Estrogen receptor agonist |
| flubendazole | -94.97 | Tubulin inhibitor |
| terreic-acid | -95.07 | BTK inhibitor |
| motesanib | -95.24 | KIT inhibitor, PDGFR receptor inhibitor, VEGFR inhibitor |
| SA-792574 | -96.68 | Microtubule inhibitor |
| CAY-10585 | -96.72 | HIF modulator |
| NPI-2358 | -97 | Tubulin inhibitor |
| vinorelbine | -97.53 | Tubulin inhibitor |
| neurodazine | -97.82 | Neurogenesis of non-pluripotent C2C12 myoblast inducer |
| peucedanin | -98.84 | Apoptosis stimulant |
